# Special Issue: Optical Characterizations of Novel Composite and Optically Active Materials

**DOI:** 10.3390/ma13225263

**Published:** 2020-11-21

**Authors:** Tatiana Perova

**Affiliations:** Department of Electronic and Electrical Engineering, Trinity College Dublin, The University of Dublin, Dublin 2, Ireland; perovat@tcd.ie; Tel.: +353-1896-1432

Market pressures have placed new demands on modern photonic and opto-electronic materials, including requirements for miniaturization, higher efficiency, tunable and controllable optical and electrical properties, and consistent performance. These demands have resulted in the design and development of a wide range of new composite materials based on nanostructures of various shapes (quantum dots, quantum wires, quantum plates) as well as on layered structures of different dimensionality (viz. one-dimensional (1D), two-dimensional (2D), and three-dimensional (2D) structures). Concurrently, the optical properties of metals and their nanoparticles attract attention due to the rapid development of plasmonics, a new and promising field of applied physics and nanotechnology. Applications of metal nanoparticles and their composites include medical diagnostics and therapeutics, drug delivery, extreme UV lasers, manipulation of light for photovoltaics, and solar energy technologies, to name but a few.

The Special Issue “Optical Characterizations of Novel Composite and Optically Active Materials” was launched to cover the latest advances in the development of novel composite materials with unique optical properties and their characterization using UV-VIS, CD, infrared, and Raman spectroscopy as well as numerical simulations. The eleven articles included in the Issue touch on different aspects of the fabrication and characterization of novel composite and nanostructured materials. A brief summary is given below.

Among the wide variety of novel nanostructured optical materials, perovskites are of particular interest thanks to their unique opto-electronic properties, such as charge carrier mobility, high photoluminescence quantum yield (PLQY), and high extinction coefficients, which can be tuned throughout the visible range by changing their chemical composition. To date, most of these materials contain lead atoms, which constitutes an obstacle to their large-scale implementation. This disadvantage can be overcome via the substitution of lead with less toxic chemical elements (e.g., Sn, Bi, Yb, etc.) and their mixtures. In a minireview by Ushakova et al. [1], research related to lead-free perovskite materials is analysed from 2016, with an emphasis on laser and lighting applications. The synthesis, chemical composition, and morphology of these materials, together with optimal device configurations, depending on the material parameters, are summarized with a focus on future challenges.

The next two papers [2,3] are devoted to the description of the advanced optical properties (in particular, strong photoluminescence) of complex composite structures based on quantum dots (QDs). Gromova et al. [2] discuss QDs of ternary metal dichalcogenides (CuInS_2_ and AgInS_2_) as promising alternatives to binary cadmium and lead-containing chalcogenide QDs. Because of their low toxicity, ternary QDs are considered suitable materials for the development of environmentally friendly and biocompatible devices. The main feature of ternary QDs is their high tolerance to defect states, resulting in broadband absorption and emission. They also exhibit giant Stokes shifts and long photoluminescence (PL) lifetimes (~100 ns). The authors of [2] focus on the investigation of the electronic structure of AgInS_2_ (AIS) and CuInS_2_ QDs. Initially, the authors provide a summary of the current state of the art in the study of the optical properties of AIS QDs and explain the basics of magnetic circular dichroism (MCD) as a tool for investigating electronic transitions in QDs. In the research section, room temperature MCD spectra of AIS/ZnS QDs are analysed together with the second derivatives of the absorption and photoluminescence excitation (PLE) spectra to provide less ambiguous information about the position of the energy transition in AIS/ZnS QDs. This study provides important insights into the electronic structure and properties of quantum nanostructures based on ternary metal dichalcogenides.

Surface passivation of lead chalcogenides with iodide atoms has spawned competition to improve the efficiency of optoelectronic devices based on QDs. Further development of QD applications requires a deeper understanding of passivation mechanisms. In the paper by Skurlov et al. [3], the authors conducted a comparative study of PbS QDs passivated with iodide, obtained in the form of colloidal solutions and solids. The authors analysed the effect of ligand exchange (LE) on the optical, electronic, and morphological properties of QD solids, as well as on the stability of the QD properties. PbI_2_-treated QDs were found to exhibit the highest PLQY and conductivity due to fewer deep-trapped states in QDs after exchange. Unfortunately, PbI_2_-treated QDs have poor environmental stability due to interaction with n-butylamine. These results indicate that careful selection of the LE procedure is required for each application.

The next papers [4,5] are devoted to improving the electro-optical characteristics of PbS QDs and hydrogenated amorphous SiC (a-SiC: H) for use in solar cells. In a study by Babaev et al. [4], an attempt was made to improve the efficiency and stability of solar cells based on colloidal PbS QDs using a functionalized reduced graphene oxide (f-rGO) interlayer. The combination of the remarkable optical properties of QDs with the exceptional electrical properties of graphene derivatives broadens the prospects for further increasing the efficiency of solar cells. The authors [4] used (3-mercaptopropyl) trimethoxysilane f-rGO to improve the active layer of QD-based solar cells. Various approaches to the implementation of functionalized rGO in the active layer of solar cells based on PbS QDs have been demonstrated. Functionalized rGO, used as intermediate layers between layers treated with tetrabutylammonium iodide, improves the fill factor and current density in PbS QD solar cells.

Vivaldo et al. [5] reported on the enhanced photoluminescence of hydrogenated amorphous silicon carbide (a-Si_1-x_C_x_:H) thin films obtained by plasma-enhanced chemical vapor deposition (PECVD). Strong PL is obtained after a fast annealing process for 60 s at temperatures of 200, 400, 600, and 800 ^o^C. These thin films are characterized by Fourier transform infrared spectroscopy (FTIR), PL spectroscopy, and energy-dispersive X-ray spectroscopy (EDS). The authors conclude that a structural rearrangement of the amorphous matrix occurs during the fast annealing process, which results in different degrees of oxidation on the a-Si_1-x_C_x_:H films. PL spectra demonstrate two emission bands: one centred in the near infrared and another in the visible range (blue peak). This study demonstrates the possibility of using these thin films in the development of optoelectronic devices, with potential application in solar cells.

Electrophoretic deposition (EPD) is an emerging technique in nanomaterial-based device fabrication. Finn et al. [6] report an in-depth study of this approach as a means to deposit colloidal quantum dots (CQDs) in a range of solvents. For the first time, they report a significant improvement of EPD performance via the use of dichloromethane for the deposition of CQDs, producing a corresponding CQD-TiO_2_ composite with a near 10-fold increase in quantum dot loading relative to that in more commonly used solvents such as chloroform or toluene. The authors propose that this effect is due to the higher dielectric constant of the solvent and therefore the stronger effect of EPD in this medium. The wide applicability of EPD as an approach to sensitise TiO_2_ electrodes is also demonstrated with a diverse range of ligand capped CdSe QDs and a range of group II-VI CQDs and group IV-VI PbS QDs.

Two-dimensional arrays of hollow nanotubes (NTs) made of TiO_2_ provide a promising platform for sensing, spectroscopy, and light harvesting applications [7]. Their simple manufacture using electrochemical anodizing, growing nanotube pillars of a finite length from Ti-foil, allows precise adjustment of the geometry and, therefore, material properties. Christian David [7] theoretically investigates these photonic crystal structures in relation to the reduction of reflection from the front surface, the achievable field enhancement, and photonic bands. Using rigorous coupled wave analysis (RCWA), he studied the optical response of photonic crystals made from thin-walled nanotubes relative to their bare Ti foil substrate, including additional charge carrier doping that might occur during growth. It is shown that arrays of TiO_2_ NTs act as photonic crystals, forming specific photonic bands. They are able to improve forward scattering—that is, to reduce reflection from the front surface—and, together with low-cost self-assembly, they represent an attractive alternative for photovoltaics and photocatalysis supported by nanostructures.

Multilayer metal-dielectric structures (screens) have long been used as bandpass electromagnetic filters in transmission or reflection modes. Electromagnetic radiation trapped within the bi-layer screens causes the temperature to rise rapidly. Walker et al. [8] analysed the temperature distribution over time for different wavelengths of incident radiation when the structures are separated from the heat sink. In these bi-layer screens, the upper (front) screen is made of a metal grid of holes, and the lower (rear) screen is made of a metal disc grid. The gap between them is filled with an array of dielectric spheres. The spheres are embedded in a dielectric base material consisting of an insulating (air, polyimide) or heat-conducting (MgO) layer. Moreover, 97% trapping of the electromagnetic intensity is achieved when the 0.15 μm gap is filled with MgO and Si spheres, which are considered as pure dielectrics—that is, without additional absorption losses. Intended applications include anti-fogging surfaces, electromagnetic shields, and energy harvesting structures.

A targeted search for promising optical media should be informed by a deep understanding of the characteristics of their interactions with light at the microscopic level. In the paper by Perova et al. [9], the dispersive local field approach was used for calculations of the microscopic optical properties of simple metals (including alkali metals) under 1D, 2D, and 3D size confinement. The microscopic dielectric functions calculated for these three cases are expressed as $\varepsilon_{2}^{mic}\left(\omega_{1D}\right)$ $\varepsilon_{2}^{mic}\left(\omega_{2D}\right)$, and $\varepsilon_{2}^{mic}\left(\omega_{3D}\right)$, respectively. It is shown that the peak positions of $\varepsilon_{2}^{mic}\left(\omega_{3D}\right)$, $\varepsilon_{2}^{mic}\left(\omega_{2D}\right)$, and $\varepsilon_{2}^{mic}\left(\omega_{1D}\right)$ spectra for simple metals, viz. alkali metals as well as Al, Be, Mg, Ga, In, Sn, and Si, are in agreement with experimental results from electron energy-loss spectroscopy and other optical techniques.

The next papers [10,11] present the results of simulations of optical properties of modern composite materials. Minkov et al. [10] employed an advanced optimizing envelope method (AOEM) for calculations of the optical characteristics of three As_x_Te_100-x_ films with different compositions, *x*, and dissimilar average thickness, *d*. Calculations were performed solely using a normal incidence interference transmittance spectrum T(λ ≤ 3000 nm) for a specimen consisting of the film on a glass substrate. A simple dual transformation of T(λ) is proposed, which is used to improve the accuracy of computation of its envelopes T_+_(λ) and T_-_(λ), accounting for the significant glass substrate absorption, especially for λ > 2500 nm. The results obtained confirm that the AOEM is capable of providing an accurate optical characterization of almost every dielectric or semiconductor film with d > 300 nm on a substrate. Li and Gao [11] applied the density functional theory (DFT) method for investigation of the adsorption of nitric oxide (NO) on undoped and Ce-doped LaCoO_3_ (011) surfaces. The main purpose of this work is to identify catalytic materials which can be used for removal of naturally occurring NO from various surfaces, as nitrous oxide is an environmental pollutant. In accordance with previous research, lanthanum-based perovskite-type oxides (e.g., LaCoO_3_) exhibit excellent catalytic activity for NO oxidation at temperatures of 300–350 °C. In addition, its catalytic activity can be enhanced significantly by modification with suitable transition metals like Ce. The DFT calculations demonstrate that the best adsorption site is unchanged after Ce doping.
